# Molecular Interplay Between the Sigma-1 Receptor, Steroids, and Ion Channels

**DOI:** 10.3389/fphar.2019.00419

**Published:** 2019-04-24

**Authors:** Sara L. Morales-Lázaro, Ricardo González-Ramírez, Tamara Rosenbaum

**Affiliations:** ^1^Departamento de Neurociencia Cognitiva, División de Neurociencias, Instituto de Fisiología Celular, Universidad Nacional Autónoma de México, Ciudad de México, Mexico; ^2^Departamento de Biología Molecular e Histocompatibilidad, Hospital General Dr. Manuel Gea González, Secretaría de Salud, Ciudad de México, Mexico

**Keywords:** Sig-1R, ion channels, steroids, NMDA – receptor, TRPV1, voltage-gated ion channel, progesterone

## Abstract

Cell excitability is tightly regulated by the activity of ion channels that allow for the passage of ions across cell membranes. Ion channel activity is controlled by different mechanisms that change their gating properties, expression or abundance in the cell membrane. The latter can be achieved by forming complexes with a diversity of proteins like chaperones such as the Sigma-1 receptor (Sig-1R), which is one with unique features and exhibits a role as a ligand-operated chaperone. This molecule also displays high intracellular mobility according to its activation level since, depletion of internal Ca^+2^ stores or the presence of specific ligands, produce Sig-1R’s mobilization from the endoplasmic reticulum toward the plasma membrane or nuclear envelope. The function of the Sig-1R as a chaperone is regulated by synthetic and endogenous ligands, with some of these compounds being a steroids and acting as key endogenous modifiers of the actions of the Sig-1R. There are cases in the literature that exemplify the close relationship between the actions of steroids on the Sig-1R and the resulting negative or positive effects on ion channel function/abundance. Such interactions have been shown to importantly influence the physiology of mammalian cells leading to changes in their excitability. The present review focuses on describing how the Sig-1R regulates the functional properties and the expression of some sodium, calcium, potassium, and TRP ion channels in the presence of steroids and the physiological consequences of these interplays at the cellular level are also discussed.

## Introduction

The Sigma-1 receptor (Sig-1R) is a protein mainly localized to the endoplasmic reticulum (ER), where it functions as a ligand-operated chaperone (Hayashi and Su, 2003, 2007). The first studies related to Sig-1R incorrectly classified it as an opioid-type receptor (Martin et al., 1976; Su, 1982), although, Sig-1R displays high affinity for (+)-benzomorphans (such as (+)-SKF 10047) and not for the negative enantiomers of these compounds (Tam, 1983; Largent et al., 1987).

**FIGURE 1:**
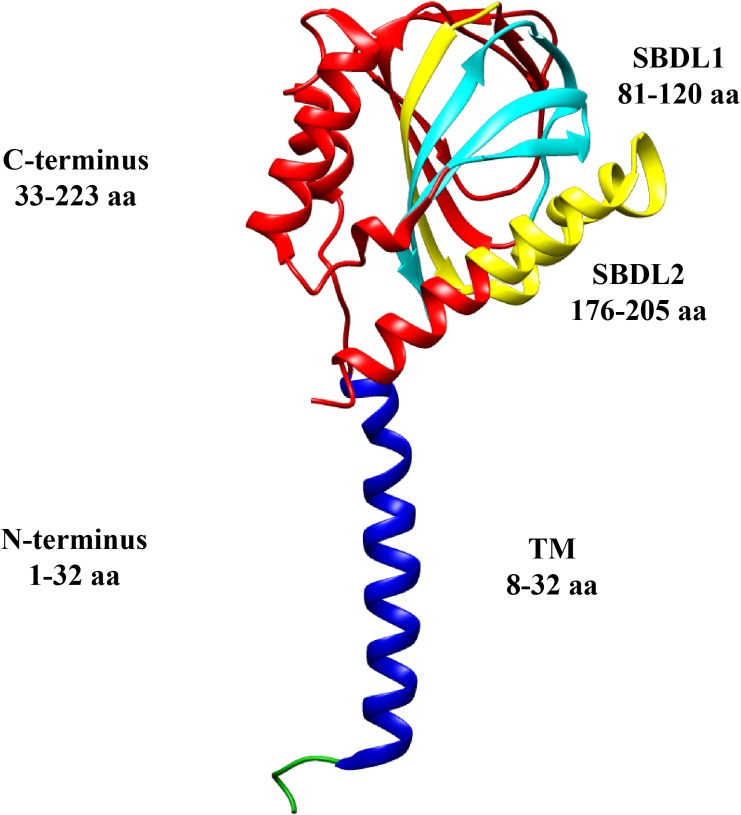
Sigma-1 receptor topology. Structural domains of a monomer of the Sig-1R are shown in different colors: N-terminus (green and blue), transmembrane segment (TM, blue), C-terminus (red), and the two Steroid Binding Domain-Like (SBDL1, aqua, and SBDL2, yellow) which are located in the C-terminus. The amino acids (aa) comprising each domain are illustrated (PDB structure entry, 5HK).

It was also hypothesized that its structure contained two transmembrane segments (Hayashi and Su, 2007; Aydar et al., 2016), however, the recent crystallographic structure for Sig-1R, shows only one transmembrane segment (Schmidt et al., 2016; Figure 1). The C-terminus of Sig-1R was also elucidated by crystallography, and it was proposed to be located facing toward the cell cytoplasm (Schmidt et al., 2016). Nonetheless, *in vivo* experiments later suggested that it is found facing the lumen of the ER (Mavylutov et al., 2018).

The high affinity of Sig-1R for dextrorotatory isomers of specific opiate benzomorphans like (+) pentazocine (Tam and Cook, 1984; Prezzavento et al., 2017), was exploited to purify it from guinea pig liver and characterize it as a ∼25 kDa protein (Hanner et al., 1996). It was determined that its sequence shares no homology with any other mammalian protein, that it contains a typical ER-retention signal within the N-terminus and, initial hydrophobicity analysis, provided the first sign of the presence of a single transmembrane segment (Hanner et al., 1996). Also, it was defined that the C-terminus of this receptor (residues 33–223) contains the ligand binding-sites (Kruse, 2017), two steroid-like binding domains (SBDL1-2) (Pal et al., 2007) and the chaperone domain (Figure 1; Hayashi and Su, 2007; Ortega-Roldan et al., 2013). Another essential feature of Sig-1R is its intracellular mobility, although it is mostly localized to the mitochondria-associated membrane (MAM) of the ER (a domain with high cholesterol content; Hayashi and Su, 2007), it still exhibits movement toward the plasma membrane and nuclear envelope (Hayashi and Su, 2003). Under basal conditions, Sig-1R forms complexes with another ER resident protein, BiP (or Gpr78) and calcium depletion from the ER derives in the dissociation of these two proteins. Then, Sig-1R becomes available to perform its chaperone functions by contributing to the stability of targets proteins, such as the inositol triphosphate (IP3) receptor (IP3R) and others, as will be here discussed (Hayashi and Su, 2007).

An important property of the function of this receptor is its regulation by synthetic and endogenous ligands (Hayashi and Su, 2004; Table 1). According to the physiological responses that these ligands elicit, they can play a role as agonists by potentiating some physiological responses or by normalizing alterations produced during some pathological conditions. On the other hand, antagonists block these effects. For example, PRE084 is considered to be a Sig-1R agonist since it improves learning impairments induced by MK-801 (a non-competitive antagonist of NMDA receptors), while this effect is suppressed by a Sig-1R antagonist, haloperidol (Maurice et al., 1994). Another example of this is the psychomotor responsiveness induced by cocaine, which is Sig-1R-dependent, an effect that is prevented by the co- or pre-administration of Sig-1R antagonists (Kourrich et al., 2013).

**Table 1:** Steroid Sig-1R ligands that regulate some ion channels.

Effect on Sig-1R	Name	Structure	Ion Channel Target
Agonist	Pregnenolone Sulfate K_i_ = 3.196 ± 0.823	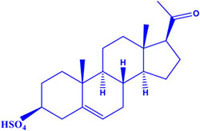	(-) L-type channels (+) NMDAr
	DHEA Sulfate K_i_ = 15.126 ± 7.69	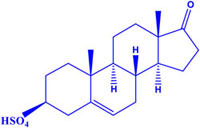	(-) Na_v_ channels (+) NMDAr
Antagonist	Progesterone K_i_ = 0.175 ± 0.55	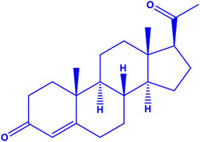	(-) TRPV1 channels (+) Na_v1.5_ channels^∗^ (-) NMDAr^∗∗^ (-) hERG channels^∗∗∗^

*K_i_ (μM) are indicated below the steroid name and represent the concentration of steroids necessary to inhibit in vitro binding of (+)-[3H]-SKF 10047 to brain homogenates (Su et al., 1988; Maurice et al., 1996). The column on the right indicates the ion channel that is the target of the steroid’s actions through modulation of Sig-1R. (+) and (-) indicate the type of effect reported. ^∗^Progesterone prevents the inhibitory effects of DMT on Nav1.5 channels. ^∗∗^Progesterone disrupts the interaction between Sig-1R and NMDAr. ^∗∗∗^This effect has not been directly demonstrated.*

It has been reported that some ligands allow for the dissociation of the Sig-1R/BiP complexes, facilitating the interaction of Sig-1R with other proteins (for example, ion channels), similar to what is described above for calcium (Hayashi and Su, 2007). Examples of ligands that promote the dissociation of Sig-1R from BiP are (+)-pentazocine, (+)-SKF 10047, PRE084, fluoxetine and cocaine (Hayashi and Su, 2007). In contrast, other Sig-1R ligands can preserve it in a silent state, either associated with BiP or by producing its oligomerization (Hayashi and Su, 2007; Mishra et al., 2015). Compounds representative of the latter are haloperidol, methamphetamine and NE-100 (Hayashi and Su, 2007; Mishra et al., 2015).

Interestingly, most of the endogenous ligands for Sig-1R are steroids (Su et al., 1988; Maurice et al., 1996). Among them are steroids synthesized in the nervous system (neurosteroids) that modulate the physiology of neuronal tissues (Corpechot et al., 1981). Neurosteroids such as pregnenolone-sulfate (PREG-S) and dehydroepiandrosterone sulfate (DHEA-S) have a role as Sig-1R agonists (Maurice et al., 1998; Table 1). Conversely, progesterone is an endogenous antagonist of this receptor, that displays the highest affinity for Sig-1R, as compared to the other steroid-type ligands (Su et al., 1988; Maurice et al., 1998; Table 1). On the other hand, testosterone, a steroid whose specific actions on Sig-1R are still unclear, only shows a partial affinity for the receptor (Su et al., 1988). In addition, cholesterol, the precursor of all steroids, can regulate Sig-1R through its binding to the C-terminus of Sig-1R (Palmer et al., 2007).

**FIGURE 2:**
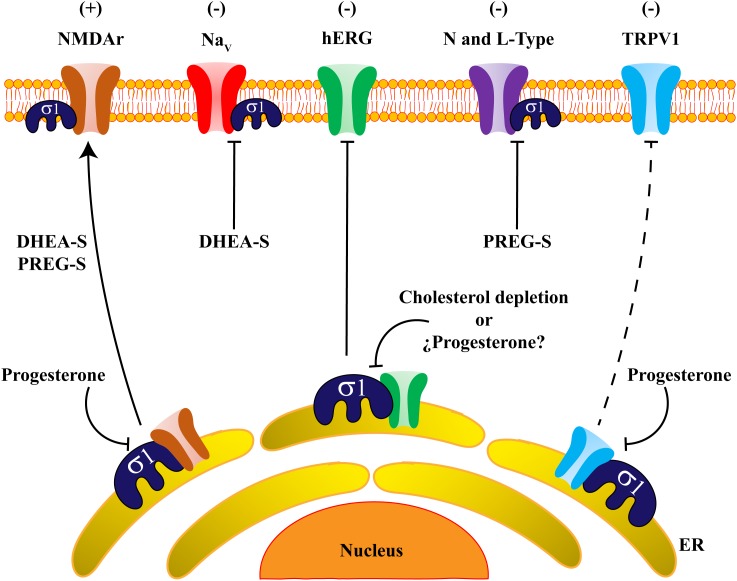
Steroids regulate ion channels through Sigma-1 receptor actions. The scheme shows an overall view of ion channels regulated by the actions of steroids on Sig-1R. DHEA-S and PREG-S positively regulate (+) NMDAr trafficking and expression, whereas progesterone disrupts the association between NMDAr and Sig-1R and blocks the positive effects of the steroids on this channel’s regulation. Otherwise, an adverse effect (-) is produced on Na^+^ persistent currents by (DHEA-S). Indeed, hERG channels form protein complexes with Sig-1R, and this association is sensitive to cholesterol depletion. Also, L-type calcium channels are negatively regulated (-) by PREG-S in a strictly Sig-1R-dependent fashion. Finally, progesterone antagonizes Sig-1R which negatively regulates (-) the TRPV1 channel, producing protein instability of TRPV1 and reducing the amount of the channel in the plasma membrane. It is possible that progesterone has the same negative effects on hERG channel-expression.

Altogether, the use of Sig-1R ligands has allowed establishing its role in neuroprotection, neurogenesis, pain, addiction, neurodegenerative and cardiovascular diseases (reviewed in Tsai et al., 2009). This review article will focus on our current understanding of how the interactions between Sig-1R and steroids regulate some ion channels such as voltage-gated potassium and sodium channels, NMDA receptors and TRP channels as well as on the resulting physiological effects of such interactions (Figure 2).

## Ion Channels

An early event of evolution was the appearance of a plasma membrane that functioned as a barrier to separate and protect the interior of cells from changes in the external conditions. This lipid barrier also allows for the separation of charges between the extracellular and intracellular regions, serving as a shelter for pore-forming proteins that allow for selective passage of ions from one side to the other which, in turn, results in the generation of a membrane potential (Hille, 2001). Some of these proteins, called voltage-gated ion channels (VGIC’s; i.e., Na_v_, K_v_, and Ca_v_), open their pores to ion conduction (sodium, potassium and calcium) in response to electrical changes, a phenomenon which is possible thanks to a voltage-sensitive domain that modulates the gating properties of these channels (Hille, 2001). Other channels are specifically activated by ligands (i.e., *N*-methyl-D-aspartate or NMDA receptors), which directly bind to certain regions of the channels modulating their gating (Hille, 2001). Also, there are polymodal ion channels, such as the Transient Receptor Potential or TRP channels, that are activated by several stimuli including those of thermal, chemical and osmotic natures (Li, 2017).

Different mechanisms can regulate the functions of ion channels that include specific pore blockers or modifiers of their gating properties (chemical compounds/toxins; Hille, 2001), post-translational modifications (phosphorylation, glycosylation) and interactions with other proteins, such as Sig-1R, among others.

Although several reports illustrate the regulation of ion channels by Sig-1R and its synthetic Sig-1R ligands, few studies have conclusively shown that endogenous Sig-1R ligands, such as steroids, modulate its interaction with some ion channels. Thus, examples of the latter will be discussed below.

## Steroid-Type Compounds as Regulators of Na_V_ Channels Through Sig-1R

Voltage-gated sodium (Na_v_) channels are molecular complexes primordial to cell depolarization in all excitable cells (Hille, 2001). They are constituted by α and β subunits (pore-forming and accessory subunits, respectively) and nine different Na_v_ channels have been identified (Na_v1.1-1.9_) (Dhar Malhotra et al., 2001).

To date, it has been demonstrated that Sig-1R interacts with Na_v1.5_ channels. These channels are expressed in the brain regulating neuronal excitation (Wu et al., 2002) and in cardiac tissue shaping the action potential in the heart (Veerman et al., 2015).

Balasuriya and collaborators demonstrated the association between Sig-1R and Na_v1.5_ channels. They performed assays using HEK293 cells transiently expressing both, Sig-1R and Na_v1.5_ channels, and determined, through atomic force microscopy (AFM) experiments, that Sig-1R directly interacts with Na_v1.5_ tetramers with a 4-fold symmetry (Balasuriya et al., 2012). This molecular association is disrupted by some of Sig-1R’s synthetic ligands (haloperidol and pentazocine).

The physiological importance of the Sig-1R interaction with Na_v1.5_ channels is exemplified in studies performed with some breast cancer cell lines, such as MDA-MB-231. This cell line constitutively expresses Sig-1R and Na_v1.5_, where it has been shown that they form a protein complex (Aydar et al., 2016). The knockdown of Sig-1R expression reduces Na_v1.5_ channels surface levels in this cancer breast cell line (Aydar et al., 2016), and the physiological consequence of this is a decrease in cell adhesion. This is a clear example of the importance of the Sig-1R/Nav1.5 protein complex in regulating the metastatic behavior of these cancer cells (Aydar et al., 2016).

Other studies have shown that the endogenous hallucinogen N,N-dimethyltryptamine (DMT) (Saavedra and Axelrod, 1972), is a ligand of Sig-1R (Fontanilla et al., 2009). DMT has been shown to reduce the activation of Na_v1.5_ channels expressed in HEK293 cells and neonatal cardiac myocytes (Fontanilla et al., 2009). Effects of this Sig-1R agonist on Na^+^ currents are strictly dependent upon Sig-1R expression since they are scarce in cells expressing low levels of Sig-1R, such as in the COS-7 cell line (Johannessen et al., 2009).

As for steroids and the roles of Sig-1R on the regulation of Na_v_ channels, it has been shown that DHEA-S negatively impacts on the function of persistent Na^+^ currents. It is known that the increase of this type of currents leads to hyperexcitability of cells expressing these channels (Deng and Klyachko, 2016). The effects of DHEA-S on persistent Na^+^ currents were evaluated in rat medial prefrontal cortex slices and were examined before and after DHEA-S perfusion (4.5 min) (Cheng et al., 2008). Whole-cell patch clamp recordings showed that DHEA-S reduces persistent Na^+^ currents an action mimicked by carbetapentane citrate (a Sig-1R agonist) and blocked by Sig-1R antagonist (i.e., haloperidol) (Cheng et al., 2008). Although a change in the overall excitability of the tissue in question would have been expected in the presence of DHEA-S, this was not observed and the reasons for this remain unclear. Nonetheless, it has been suggested by the authors of this study that, such a lack of change in the tissue’s excitability in the presence of DHEA-S, may be due to a positive effect of this compound on other molecular targets. This would lead to neutralization of the negative regulation of persistent Na^+^ currents by DHEA-S and a neutral effect on neuronal excitability (Cheng et al., 2008).

Moreover, it has been suggested that the regulation of persistent Na^+^ currents by DHEA-S, is probably relevant under pathological conditions such as brain ischemia. Under this scenario, negative regulation of persistent Na^+^ currents by DHEA-S, through the actions of Sig-1R, would lead to a decrease in neuronal excitability, resulting in a neuroprotective effect. It has been proposed that DHEA-S may be a desirable candidate for therapeutic approaches directed toward relieving induced cerebral ischemia infarction (Yabuki et al., 2015).

In summary, there are only a few evidences showing the direct relationship between Sig-1R, steroids and Na_v_ channels. Nonetheless, available studies suggest that the negative role of synthetic Sig-1R ligands on Na^+^ currents are similar to those promoted by steroidal Sig-1R agonists (i.e., DHEA-S) (Figure 2).

## K_V_ Channels Are Functional Targets of the Actions of Cholesterol on Sig-1R

The generation of action potentials depends upon a fine-tuned coordination of different electrical phases, among which is repolarization. Through this process, the membrane potential is returned to negative voltage values to ensure that an excitable cell can respond to new stimuli. For repolarization to occur, the activation of voltage-gated K^+^ channels (K_v_) is essential (Hille, 2001; Kandel et al., 2012).

It has been shown that Sig-1R forms complexes with these ion channels regulating their function or abundance in the plasma membrane. Ligands of Sig-1R highly regulate the formation of some of these complexes. For instance, coimmunoprecipitation assays have demonstrated that Sig-1R is associated to Kv_1.4_ in posterior pituitary nerve terminals from rat and also in Xenopus oocytes with heterologous expression of Sig-1R and K_v1.4_ channels (Aydar et al., 2002). In the latter, whole-cell recordings showed that (+)-SKF 10047, downregulates K_v1.4_ channel outward currents, indicating a negative role of Sig-1R on the function of these proteins (Aydar et al., 2002).

Conversely, cocaine triggers the dissociation of Sig-1R from BiP, leading to its interaction with K_v1.2_ and facilitating channel translocation to the plasma membrane. As a result of this, K_v1.2_ function is positively regulated, resulting in neuronal hypoactivity (Kourrich et al., 2013). These results highlight the physiological consequence of a cocaine-induced long-lasting association of these two proteins by which, an enduring experience-dependent plasticity phenomenon, occurs. This also pinpoints a mechanism that can shape neuronal and behavioral responses to cocaine, as suggested by Kourich and collaborators.

Despite the lack of direct experimental evidences showing the effects of steroids on the association of Sig-1R with K_v_ channels, some reports have demonstrated a possible interplay between them. For example, patch clamp experiments performed in the K562 leukemic cell line, which endogenously expresses Sig-1R and hERG channels (a K_v_ channel also expressed in cardiac tissues), showed that hERG currents are inhibited by igmesine and (+) pentazocine (both of which are Sig-1R ligands). In addition, silencing of Sig-1R using shRNA-based strategies, also demonstrated reduced hERG current-densities without affecting hERG-channel transcription, but rather by decreasing the amount of the mature form of the channel on the plasma membrane of the cells (Crottes et al., 2011).

It has also been reported that progesterone alters hERG-channel expression. By using HEK293 cells stably-expressing hERG channels and whole-cell voltage clamp recordings, it has been demonstrated that progesterone decreases hERG current-density. This effect was also observed in an hERG-channel endogenous expression system of rat neonatal cardiac myocytes (Wu et al., 2011). Confocal microscopy and western blot analysis of surface proteins showed that progesterone decreases the amount of the mature form of hERG-channel proteins in the plasma membrane and induces channel accumulation in the ER. Moreover, using filipin cell-staining techniques, it was shown that treatment with progesterone produces disruption of cholesterol homeostasis, impairing adequate hERG-channel folding and traffic to the Golgi compartment (Wu et al., 2011). Since progesterone is a Sig-1R antagonist, it could be hypothesized that these effects are produced through a disruption of the Sig-1R and hERG protein complexes. Alternatively, this could be the result of an alteration of cholesterol homeostasis, affecting the function or localization of Sig-1R. The consequences of both possibilities are an improper folding and traffic of hERG channels; thus, the overall effect of progesterone is negative regulation of channel expression in the plasma membrane (Figure 2).

Additionally, AFM imaging and homogenous time-resolved fluorescence experiments, have demonstrated that Sig-1R interacts with hERG channels with a 4-fold symmetry in the plasma membrane of HEK293 cells stably transfected with both proteins (Balasuriya et al., 2014). This is a similar scenario to the that reported for the formation of protein complexes between Sig-1R and Na_v1.5_ channels (Balasuriya et al., 2012). Interestingly, this association is independent of Sig-1R’s ligands but susceptible to cholesterol depletion (Balasuriya et al., 2014). Accordingly, it has been suggested that Sig-1R ligands can displace cholesterol from its binding site altering the distribution of the receptor in the cell and profoundly impacting on its association with other proteins (Palmer et al., 2007).

This experimental evidence suggests that Sig-1R supports a suitable assembly and folding of immature hERG channels in order to enable them to exit from the ER. Thus, it follows that progesterone and cholesterol affect Sig-1R actions and reduce hERG channel maturation.

Similar results have been obtained for SK3 channels, the small-conductance calcium-activated potassium channels, for which expression of these proteins is regulated by the cellular content of Sig-1R and cholesterol (Gueguinou et al., 2017). The molecular silencing of Sig-1R or the use of igmesine (a Sig-1R ligand) decreases the amount of SK3 channels localized to lipid-enriched nanodomains in breast cells. This, in turn, results in a reduction in the migration of these cancer cells (Gueguinou et al., 2017). Thus, these findings emphasize an interesting area of research in which, the regulation of Sig-1R activity, may be an alternative to control the metastatic potential of certain types of cancers where the levels of Sig-1R are upregulated (i.e., colorectal or breast cancers) (Gueguinou et al., 2017).

## Regulation of Ca_V_ Channels Through Sig-1R’s Activation

Voltage-gated calcium channels (Ca_v_) are the main transducers of membrane potential changes (Hille, 2001). Their activation produces the influx of Ca^+2^ ions to the cell, where they function as second messengers to activate different cell signaling pathways, leading to diverse physiological consequences. Ca_v_ channels are constituted by pore-forming subunits, α_1_ (similar to Na_v_ channels) and by accessory subunits (α_2_δ, β, and γ), which are necessary for a suitable function and expression of these channels. According to the types of Ca^+2^ currents that they generate, these proteins are classified as L-, N-, P/Q-, R-, and T-type calcium channels (reviewed in Catterall, 2000).

So far, there is interesting evidence about the interactions between Ca_v_ channels and Sig-1R. In this respect, the data show that Sig-1R activation by different synthetic agonists, negatively influences Ca_v_ channels functions, as shown in isolated intracardiac neurons of neonatal rats (Zhang and Cuevas, 2002). In this experimental model, Ca_v_ channel inactivation rates are increased, and the steady-state voltage-dependences of activation and inactivation are shifted to negative potentials.

The adverse effects of Sig-R ligands on Ca_v_ channel function have also been observed in cholinergic interneurons from the rat striatum. Here, agonists of Sig-1R such as (+)-SKF 10047 and PRE-084, inhibit N-type calcium currents and, as it would be expected, BD1047, a Sig-1R antagonist obliterates this phenomenon. FRET and coimmunoprecipitation experiments demonstrated that N-type channels and Sig-1R form protein-complexes when these proteins are expressed in HEK293 cells. The authors of this study proposed that Sig-1R agonists produce a conformational change in these protein complexes that negatively regulates N-type channels (Zhang et al., 2017).

Likewise, the negative roles of Sig-1R ligands on Ca_v_ channels have been observed in primary neuronal cultures from the hippocampus, where SA4503 (a Sig-1R agonist), inhibits N- and L-Type currents, producing an increase in axonal outgrowth (Li et al., 2017).

As for L-type ion channels, these have been shown to co-localize with Sig-1R in retinal ganglion cells (Mueller et al., 2013). Likewise, physical interactions between these proteins have also been demonstrated through coimmunoprecipitation assays in these cells (Tchedre et al., 2008).

Furthermore, an interplay between L-Type channels, Sig-1R and the neurosteroid, PREG-S, has been reported (Sabeti et al., 2007). This was evaluated using electrophysiological recordings from CA1 neurons of rat hippocampal slices perfused with PREG-S before and during the induction of long-term potentiation (LTP) of excitatory transmission. The data demonstrated that LTP increased in slices subjected to PREG-S perfusion. Suitably, nimodipine, an antagonist of L-type calcium channels, blocked PREG-S-induced LTP. Additionally, perfusion of PREG-S and BD1047, also blocked LTP in hippocampal slices, strongly supporting the role of PREG-S acting through Sig-1R in this process (Sabeti et al., 2007). Thus, in this neuronal context, regulation of the function of L-type channels confers synaptic plasticity (Figure 2).

## Regulation of Nmda Receptors by Neurosteroid Molecules and Sig-1R

The *N*-methyl-D-aspartate receptor (NMDAr) is a heterotetrameric ionotropic receptor formed by the assembly of two NR1 and two NR2 or NR3 subunits. NMDAr functions as a channel permeable to Ca^+2^, its activation is produced by the binding of two different ligands (glutamate and glycine), profoundly impacting on neuronal plasticity, memory and learning processes (reviewed in Hansen et al., 2018).

Regulation of NMDAr by Sig-1R ligands has been extensively reported and, positive effects on their function, strongly correlate to Sig-1R’s activation. A pioneering study by Monnet and collaborators demonstrated that a synthetic Sig-1R agonist potentiated neuronal activation induced by NMDAr in CA3 dorsal hippocampal neurons, an effect that was reverted by haloperidol (Monnet et al., 1990). Positive NMDAr regulation by Sig-1R’s agonists leads to an improvement in learning and memory since it has been shown that PRE084, attenuates the impairment of learning in mice treated with an NMDAr antagonist (Maurice et al., 1994). A recent study showed that memory deficits produced in mice where brain ischemia was induced, were improved by the use of Sig-1R agonists while they were worsened by antagonism of the NR2 subunits (Xu et al., 2017).

**FIGURE 3:**
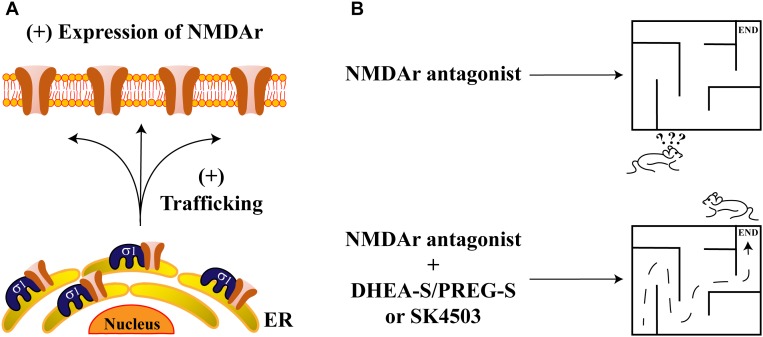
Sigma-1 receptor and its endogenous ligands improve memory deficits. **(A)** The scheme represents the protein-complex between Sig-1R and NMDAr, which enhances the channel’s trafficking to the plasma membrane. **(B)** NMDAr antagonism produces deficits in memory and spatial work (upper panel). Sig-1R’s agonists such as DHEA-S, PREG-S, or SA4503, ameliorate memory impairments due to NMDAr antagonism (lower panel).

Recently, a direct interaction between Sig-1R and the NR1 subunit of NMDAr has been revealed *in vitro* through the use of AFM imaging. Proximity-ligation assays also confirmed this interaction, supporting an *in vivo* association between these proteins (Balasuriya et al., 2013). This protein-complex is disrupted by some Sig-1R ligands such as BD1063, cannabidiol and progesterone, as demonstrated by pull-down assays (Rodriguez-Munoz et al., 2018). In addition, it has also been shown that the NR2 subunit of NMDAr is positively regulated by Sig-1R agonists, producing an upregulation in NR2-protein-expression and increasing traffic of NR2 to the plasma membrane (Pabba et al., 2014; Figure 3A). Finally, it has been shown that Sig-1R knockout mice display decreases in NMDAr-mediated currents and that these animals exhibit deficiencies in neurogenesis at the hippocampal dentate gyrus (Sha et al., 2013). These data suggest that Sig-1R activation positively influences NMDAr function during memory and learning processes.

Effects of steroid-type Sig-1R ligands on NMDAr functions have also been reported. For example, rats subjected to intraperitoneal injection of dizocilpine, (a competitive antagonist of NMDAr), exhibit spatial working and memory deficits. These effects are attenuated by DHEA-S and PREG-S or by a SA4503, all of which are Sig-1R agonists (Figure 3B). In contrast, progesterone or NE-100 (both of which constitute Sig-1R antagonists), block the ameliorative effects of DHEA-S and PREG-S on dizocilpine-induced memory deficits (Zou et al., 2000).

Similarly, some brain ischemia animal models display impairment of LTP at the hippocampal CA1 area, an effect that is prevented by DHEA-S. On the contrary, NE100 and progesterone revert the positive actions of DHEA-S in this model (Li et al., 2006). Finally, the protective effects of DHEA-S on spatial memory have also been reported and have demonstrated that they are dependent upon Sig-1R’s actions (Li et al., 2009).

## Trpv1: the First Trp Channel Shown to Be Regulated by Sig-1R

Transient Receptor Potential (TRP) ion channels allow for the influx of cations in a non-selective fashion. These proteins possess four subunits, giving rise to functional tetramers (reviewed in Ramsey et al., 2006). According to their structural features, these channels have been classified into seven subfamilies: TRPA (ankyrin), TRPC (canonical), TRPM (melastatin), TRPML (mucolipin), TRPN (no-mechanoreceptor potential C), TRPP (polycystic) and TRPV (vanilloid) (reviewed in Li, 2017). Several of the members of these subfamilies are implicated in the transduction of thermal, chemical and osmotic stimuli.

TRPV1 channels have been extensively studied for their roles in pain. They are mainly expressed by nociceptors (Aδ and C-Fibers) where they are essential for the transduction of noxious signals. These channels are activated by high temperatures (≥ 42°C), irritant compounds (capsaicin, resiniferatoxin, allicin, etc.) (Caterina et al., 1997; Salazar et al., 2008) and by changes in extra- and intracellular pH (acid and basic, respectively) (Caterina et al., 1997; Jordt et al., 2000; Dhaka et al., 2009). Additionally, TRPV1’s activation can also be achieved by some endogenous compounds released during inflammation or tissue injury, such as lysophosphatidic acid (LPA) (Nieto-Posadas et al., 2011), among other endogenous agonists (reviewed in Morales-Lazaro et al., 2013). Since TRPV1 exhibits a pivotal role in painful signal transduction, this channel has become a pharmacological target with several research groups around the world focusing on ways to regulate its function to relieve certain types of pain.

So far, several synthetic compounds and some of a natural origin (i.e., oleic acid) (Morales-Lazaro et al., 2016), have been described as negative regulators of TRPV1’s activation. However, few studies have revealed alternative ways to regulate TRPV1’s functions, including manipulating the abundance of this channel in the plasma membrane of nociceptors. Just recently, our group described that Sig-1R is a crucial molecular target that can regulate the amount of TRPV1 channels localized to the plasma membrane of cells, without affecting channel transcription (Ortiz-Renteria et al., 2018). This constituted the first report for a TRP channel as being regulated by Sig-1R, highlighting the role of a direct interaction between TRPV1 and Sig-1R in pain.

In this study, we found that a synthetic ligand of Sig-1R, BD1063, decreased TRPV1 protein levels in mice dorsal root ganglia (DRG) and HEK293 cells transiently expressing TRPV1. This effect was mimicked by the knockdown of Sig-1R expression in TRPV1-expressing HEK293 cells. Furthermore, progesterone also produced down-regulation of TRPV1 expression, as demonstrated by western blot assays. Worth noting is that the effects of progesterone on TRPV1 expression were found to be independent of its classical nuclear receptors (Ortiz-Renteria et al., 2018).

Also, whole-cell recordings showed that Sig-1R knockdown and the addition of BD1063 or progesterone to cell cultures, reduced the current-densities evoked by capsaicin, indicating that negative regulation of Sig-1R (either by reducing Sig-1R expression or using its antagonists), decreased the amount of TRPV1 localized to the plasma membrane. This negative effect on TRPV1 expression was prevented through the inhibition of proteasomal degradation, suggesting that Sig-1R is necessary for TRPV1 protein stability and confirms an independent effect of a transcriptional mechanism (Ortiz-Renteria et al., 2018).

**FIGURE 4:**
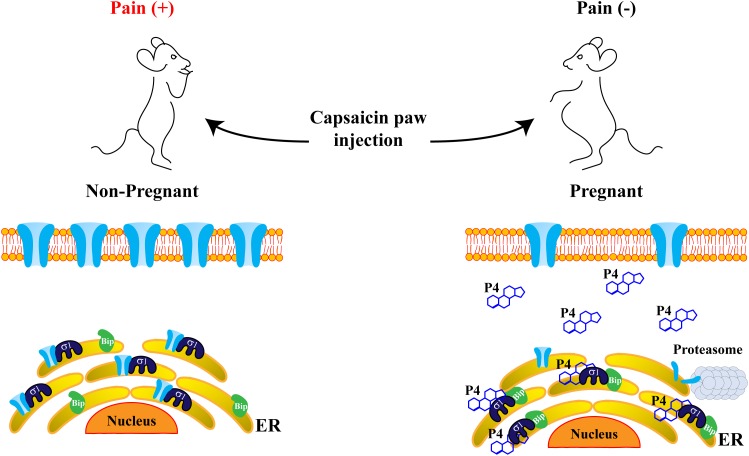
Progesterone attenuates pain-related behavior through disruption of the Sig-1R/TRPV1 complex. Sig-1R and TRPV1 channels are physically associated in the endoplasmic reticulum (ER), improving TRPV1 stability and resulting in suitable TRPV1 expression in the plasma membrane, where the channel transduces painful signals. Consequently, non-pregnant mice display pain behaviors in response to capsaicin paw-injection (left). However, in pregnant mice (right), when progesterone levels considerably increase, the formation of the complex between Sig-1R and the Binding immunoglobulin protein (BiP), is promoted. This maintains Sig-1R in a sequestered state and blocks its association with the TRPV1 channel, improving protein instability and avoiding degradation through the proteasomal pathway. Thus, TRPV1 protein levels in the plasma membrane decrease, leading to an increased pain threshold in response to capsaicin in pregnant mice.

Besides, we established a direct association between Sig-1R and the TRPV1 channel through coimmunoprecipitation and FRET experiments. We found that Sig-1R interacts with the transmembrane domain of TRPV1, similarly, to what had been previously reported for K_v1.3_ ion channels (Kinoshita et al., 2012). Interestingly, this protein-complex was sensitive to Sig-1R ligands, since BD1063 and progesterone decreased the association between TRPV1 and Sig-1R. Furthermore, by confocal microscopy analysis, we observed that this protein-protein association is most prominent at the ER compartment and occurs less at the level of the plasma membrane. Together, all of these evidences pointed to a role of Sig-1R in conferring protein stability during the biogenesis of the channel, with Sig-1R preventing the misfolding of TRPV1 and avoiding its degradation by the proteasomal system (Figure 4).

Since, progesterone is the steroid with higher affinity for Sig-1R, its interactions with this receptor and the physiological consequences of such interplays have been a subject of focus in pregnancy, a physiological phase where progesterone levels are high (Bergeron et al., 1999). With this in mind, we explored if pain thresholds in response to capsaicin were different between non- and pregnant mice. The results indicated that, during pregnancy, pain-like behavior in response to the TRPV1 agonist was significantly decreased in mice, as compared to their non-pregnant counterparts (Figure 4). This led us to conclude that elevated levels of progesterone, such as those found during pregnancy, could confer protection against painful situations through the disruption of the protein complex between Sig-1R and TRPV1. This, in turn, would result in the downregulation of TRPV1’s levels in the plasma membrane, ultimately decreasing the pain threshold associated with TRPV1’s activation (Figure 4).

Several sources in the literature, together with our findings, have highlighted Sig-1R as a protein widely associated with nociception. For example, it has been reported that Sig-1R knockout mice, exhibit endurance to pain and mechanical allodynia induced by formalin and capsaicin, respectively (Cendan et al., 2005; Entrena et al., 2009).

Since Sig-1R is expressed in DRG neurons (Mavlyutov et al., 2016) and in specific regions of the spinal cord (Alonso et al., 2000), its actions are relevant at the pre and post-synaptic levels (reviewed in Romero et al., 2016). Thus, its roles in pain responses must involve more than one molecular target. For instance, several reports show that Sig-1R agonists induce and maintain central sensitization during painful states (Romero et al., 2012; Entrena et al., 2016; Choi et al., 2017). Some of Sig-1R’s effects are through the upregulation or phosphorylation of the NR1 subunit of the NMDAr that lead to neuronal overexcitability (Roh et al., 2010). Conversely, antagonists of Sig-1R prevent or decrease pain in some neuropathic pain animal models since these compounds inhibit the upregulation of NR1 (Zhu et al., 2015). Besides it has been demonstrated that DHEA-S and PREG-S positively regulate the activity of P2X receptors resulting in the potentiation of the mechanical allodynia induced through these receptors (Kwon et al., 2016).

In conclusion, Sig-1R is essential for regulating peripheral and central sensitization by interacting with various molecular targets such as TRPV1 channels, P2X channels, NMDAr and it is possible that several other proteins involved in these processes, will be identified in the future.

### Progesterone as a Prototypical Endogenous Ligand of Sig-1R

Up to this point, we have emphasized findings that link the actions of Sig-1R and steroids to the function of ion channels. It is important to stress that, the steroid concentrations used in most of the experiments here described, are well above the reported physiological range.

The low affinity of steroids to Sig-1R justifies the use of high concentrations of steroid ligands in different experimental systems. For example, a study by Su et al. (1988) showed that progesterone competes (Ki = 268 nM) with [H3](+)-SKF 10047 for the binding site in Sig-1R and exhibits higher affinity for this receptor. However, other steroids are required at much higher concentrations than those of progesterone to displace [H3](+)-SKF 10047 (Su et al., 1988). Nevertheless, this progesterone concentration is still too high to be normally circulating in blood but, in pregnancy, the circulating levels os this steroids can rise to 400 nM (Neill et al., 1969), reaching concentrations high enough to regulate Sig-1R.

In agreement with the results obtained by Su et al. (1988), Maurice et al. (1996) showed that progesterone displaces the binding of (+)-SKF 10047 to rat brain homogenates with a Ki of 175 nM. They showed that [H3](+)-SKF 10047 binding is significantly reduced in the hippocampus and the cortex from pregnant mice, as compared to non-pregnant female or male mice (Maurice et al., 1996).

These findings, together with ours where we showed that pregnant female mice display high pain-thresholds as compared to unpregnant mice (Ortiz-Renteria et al., 2018), suggesting that high circulating progesterone levels in pregnancy are enough to modify pain thresholds in mice.

Recently, a new high-affinity Sig-1R selective radiotracer ([^18^F] FPS) has been developed. This compound has been used to perform PET studies in Rhesus monkeys showing high uptake of the radiotracer in brain regions where there are moderate densities of Sig-1R (Mach et al., 2005). Interestingly, animals pre-injected with progesterone displayed a significant reduction in the uptake of the radiotracer in the brain of the monkeys, confirming that progesterone displaces the radiotracer from Sig-1R’s binding sites (Mach et al., 2005). In addition, this radiotracer was used to perform *in vitro* binding assays in rat forebrain homogenates showing that progesterone inhibits the binding of the radiotracer to Sig-1R. Notably, the Ki for progesterone is of 36 nM, a concentration of progesterone achieved during the luteal phase of the menstrual cycle (Neill et al., 1969). This finding further strengthens the notion that low progesterone levels also play a role in the activity of Sig-1R and that this steroid functions as an endogenous ligand of this receptor (Waterhouse et al., 2007).

## Conclusion

Regulation of ion channel physiology has been a subject of intense research for several decades. The implications of such modifications of ion channel function are extremely diverse and important, and they are exemplified by the roles that these proteins play under normal physiological conditions but also in pathophysiological scenarios. Not only do these essential proteins regulate muscle contraction, neuronal excitability, and hormonal secretion but changes in their expression and function during pathological conditions lead to severe diseases such as epilepsy, diabetes, ataxia, pain, itch, etc. For many years, studies have focused on identifying molecular targets of synthetic and naturally-produced agents to regulate the activity of ion channels.

In addition to the identification of chemicals that specifically bind to members of different ion channel families to alter their biophysical properties, research groups have also focused on determining how protein-protein interactions regulate these. Among the proteins that can bind and change ion channel function and expression are chaperones and their roles and the consequences of their misfunctions are being studied in several diseases (Broadley and Hartl, 2009). The Sig-1R has been shown to play essential roles for the adequate function of several types of cells (Tsai et al., 2009), including those that are electrically excitable and hence express ion channels. Thus, from these studies and those discussed here, it is evident that a mechanism by which we may regulate ion channel physiology is through tools that allow us to manipulate the interactions between Sig-1R and these other proteins if this was to be possible without other severe consequences. But utmost important is that, by studying the interactions of Sig-1R with ion channels, we have gained valuable knowledge on how this receptor regulates ion channels. In turn, this has also helped us understand the physiological consequences of modifying the interplays between Sig-1R and ion channels for the function of the cells where these proteins are expressed.

## Author Contributions

All authors listed have made a substantial, direct and intellectual contribution to the work, and approved it for publication.

## Conflict of Interest Statement

The authors declare that the research was conducted in the absence of any commercial or financial relationships that could be construed as a potential conflict of interest.

## References

[B1] Alonso G. Phan V. Guillemain I. Saunier M. Legrand A. Anoal M. (2000). Immunocytochemical localization of the sigma(1) receptor in the adult rat central nervous system. *Neuroscience* 97 155–170. 10771347 10.1016/s0306-4522(00)00014-2

[B2] Aydar E. Palmer C. P. Klyachko V. A. Jackson M. B. (2002). The sigma receptor as a ligand-regulated auxiliary potassium channel subunit. *Neuron* 34 399–410. 11988171 10.1016/s0896-6273(02)00677-3

[B3] Aydar E. Stratton D. Fraser S. P. Djamgoz M. B. Palmer C. (2016). Sigma-1 receptors modulate neonatal Nav1.5 ion channels in breast cancer cell lines. *Eur. Biophys. J.* 45 671–683. 27160185 10.1007/s00249-016-1135-0

[B4] Balasuriya D. D’Sa L. Talker R. Dupuis E. Maurin F. Martin P. (2014). A direct interaction between the sigma-1 receptor and the hERG voltage-gated K^+^ channel revealed by atomic force microscopy and homogeneous time-resolved fluorescence (HTRF^(R)^). *J. Biol. Chem.* 289 32353–32363. 10.1074/jbc.M114.603506 25266722 PMC4231707

[B5] Balasuriya D. Stewart A. P. Crottes D. Borgese F. Soriani O. Edwardson J. M. (2012). The sigma-1 receptor binds to the Nav1.5 voltage-gated Na^+^ channel with 4-fold symmetry. *J. Biol. Chem.* 287 37021–37029. 10.1074/jbc.M112.382077 22952230 PMC3481303

[B6] Balasuriya D. Stewart A. P. Edwardson J. M. (2013). The sigma-1 receptor interacts directly with GluN1 but not GluN2A in the GluN1/GluN2A NMDA receptor. *J. Neurosci.* 33 18219–18224. 10.1523/JNEUROSCI.3360-13.2013 24227730 PMC3828470

[B7] Bergeron R. de Montigny C. Debonnel G. (1999). Pregnancy reduces brain sigma receptor function. *Br. J. Pharmacol.* 127 1769–1776. 10.1038/sj.bjp.0702724 10482906 PMC1566170

[B8] Broadley S. A. Hartl F. U. (2009). The role of molecular chaperones in human misfolding diseases. *FEBS Lett.* 583 2647–2653. 10.1016/j.febslet.2009.04.029 19393652

[B9] Caterina M. J. Schumacher M. A. Tominaga M. Rosen T. A. Levine J. D. Julius D. (1997). The capsaicin receptor: a heat-activated ion channel in the pain pathway. *Nature* 389 816–824. 10.1038/39807 9349813

[B10] Catterall W. A. (2000). Structure and regulation of voltage-gated Ca^2+^ channels. *Annu. Rev. Cell Dev. Biol.* 16 521–555. 10.1146/annurev.cellbio.16.1.521 11031246

[B11] Cendan C. M. Pujalte J. M. Portillo-Salido E. Montoliu L. Baeyens J. M. (2005). Formalin-induced pain is reduced in sigma(1) receptor knockout mice. *Eur. J. Pharmacol.* 511 73–74. 10.1016/j.ejphar.2005.01.036 15777781

[B12] Cheng Z. X. Lan D. M. Wu P. Y. Zhu Y. H. Dong Y. Ma L. (2008). Neurosteroid dehydroepiandrosterone sulphate inhibits persistent sodium currents in rat medial prefrontal cortex via activation of sigma-1 receptors. *Exp. Neurol.* 210 128–136. 10.1016/j.expneurol.2007.10.004 18035354

[B13] Choi S. R. Moon J. Y. Roh D. H. Yoon S. Y. Kwon S. G. Choi H. S. (2017). Spinal D-serine increases PKC-dependent GluN1 phosphorylation contributing to the sigma-1 receptor-induced development of mechanical allodynia in a mouse model of neuropathic pain. *J. Pain* 18 415–427. 10.1016/j.jpain.2016.12.002 27986591

[B14] Corpechot C. Robel P. Axelson M. Sjovall J. Baulieu E. E. (1981). Characterization and measurement of dehydroepiandrosterone sulfate in rat brain. *Proc. Natl. Acad. Sci. U.S.A.* 78 4704–4707. 6458035 10.1073/pnas.78.8.4704 PMC320231

[B15] Crottes D. Martial S. Rapetti-Mauss R. Pisani D. F. Loriol C. Pellissier B. (2011). Sig1R protein regulates hERG channel expression through a post-translational mechanism in leukemic cells. *J. Biol. Chem.* 286 27947–27958. 10.1074/jbc.M111.226738 21680736 PMC3151040

[B16] Deng P. Y. Klyachko V. A. (2016). Increased persistent sodium current causes neuronal hyperexcitability in the entorhinal cortex of fmr1 knockout mice. *Cell Rep.* 16 3157–3166. 10.1016/j.celrep.2016.08.046 27653682 PMC5055130

[B17] Dhaka A. Uzzell V. Dubin A. E. Mathur J. Petrus M. Bandell M. (2009). TRPV1 is activated by both acidic and basic pH. *J. Neurosci.* 29 153–158. 10.1523/JNEUROSCI.4901-08.2009 19129393 PMC2729567

[B18] Dhar Malhotra J. Chen C. Rivolta I. Abriel H. Malhotra R. Mattei L. N. (2001). Characterization of sodium channel alpha- and beta-subunits in rat and mouse cardiac myocytes. *Circulation* 103 1303–1310. 11238277 10.1161/01.cir.103.9.1303

[B19] Entrena J. M. Cobos E. J. Nieto F. R. Cendan C. M. Gris G. Del Pozo E. (2009). Sigma-1 receptors are essential for capsaicin-induced mechanical hypersensitivity: studies with selective sigma-1 ligands and sigma-1 knockout mice. *Pain* 143 252–261. 10.1016/j.pain.2009.03.011 19375855

[B20] Entrena J. M. Sanchez-Fernandez C. Nieto F. R. Gonzalez-Cano R. Yeste S. Cobos E. J. (2016). Sigma-1 receptor agonism promotes mechanical allodynia after priming the nociceptive system with capsaicin. *Sci. Rep.* 6:37835. 10.1038/srep37835 27886264 PMC5122889

[B21] Fontanilla D. Johannessen M. Hajipour A. R. Cozzi N. V. Jackson M. B. Ruoho A. E. (2009). The hallucinogen N,N-dimethyltryptamine (DMT) is an endogenous sigma-1 receptor regulator. *Science* 323 934–937. 10.1126/science.1166127 19213917 PMC2947205

[B22] Gueguinou M. Crottes D. Chantome A. Rapetti-Mauss R. Potier-Cartereau M. Clarysse L. (2017). The SigmaR1 chaperone drives breast and colorectal cancer cell migration by tuning SK3-dependent Ca^2+^ homeostasis. *Oncogene* 36 3640–3647. 10.1038/onc.2016.501 28114279

[B23] Hanner M. Moebius F. F. Flandorfer A. Knaus H. G. Striessnig J. Kempner E. (1996). Purification, molecular cloning, and expression of the mammalian sigma1-binding site. *Proc. Natl. Acad. Sci. U.S.A.* 93 8072–8077. 8755605 10.1073/pnas.93.15.8072 PMC38877

[B24] Hansen K. B. Yi F. Perszyk R. E. Furukawa H. Wollmuth L. P. Gibb A. J. (2018). Structure, function, and allosteric modulation of NMDA receptors. *J. Gen. Physiol.* 150 1081–1105. 10.1085/jgp.201812032 30037851 PMC6080888

[B25] Hayashi T. Su T. P. (2003). Sigma-1 receptors (sigma(1) binding sites) form raft-like microdomains and target lipid droplets on the endoplasmic reticulum: roles in endoplasmic reticulum lipid compartmentalization and export. *J. Pharmacol. Exp. Ther.* 306 718–725. 10.1124/jpet.103.051284 12730355

[B26] Hayashi T. Su T. P. (2004). Sigma-1 receptor ligands: potential in the treatment of neuropsychiatric disorders. *CNS Drugs* 18 269–284. 15089113 10.2165/00023210-200418050-00001

[B27] Hayashi T. Su T. P. (2007). Sigma-1 receptor chaperones at the ER-mitochondrion interface regulate Ca^2+^ signaling and cell survival. *Cell* 131 596–610. 10.1016/j.cell.2007.08.036 17981125

[B28] Hille B. (2001). *Ionic Channels of Excitable Membranes.* Sunderland, MA: Sinauer.

[B29] Johannessen M. Ramachandran S. Riemer L. Ramos-Serrano A. Ruoho A. E. Jackson M. B. (2009). Voltage-gated sodium channel modulation by sigma-receptors in cardiac myocytes and heterologous systems. *Am. J. Physiol. Cell Physiol.* 296 C1049–C1057. 10.1152/ajpcell.00431.2008 19279232 PMC2681379

[B30] Jordt S. E. Tominaga M. Julius D. (2000). Acid potentiation of the capsaicin receptor determined by a key extracellular site. *Proc. Natl. Acad. Sci. U.S.A.* 97 8134–8139. 10.1073/pnas.100129497 10859346 PMC16682

[B31] Kandel E. R. Schwartz J. H. Jessell T. M. Siegelbaum S. A. (2012). *Principles of Neural Science*, 5th Edn. New York, NY: McGraw-Hill Education.

[B32] Kinoshita M. Matsuoka Y. Suzuki T. Mirrielees J. Yang J. (2012). Sigma-1 receptor alters the kinetics of Kv1.3 voltage gated potassium channels but not the sensitivity to receptor ligands. *Brain Res.* 1452 1–9. 10.1016/j.brainres.2012.02.070 22433979 PMC3670091

[B33] Kourrich S. Hayashi T. Chuang J. Y. Tsai S. Y. Su T. P. Bonci A. (2013). Dynamic interaction between sigma-1 receptor and Kv1.2 shapes neuronal and behavioral responses to cocaine. *Cell* 152 236–247. 10.1016/j.cell.2012.12.004 23332758 PMC4159768

[B34] Kruse A. (2017). Structural insights into sigma1 function. *Handb. Exp. Pharmacol.* 244 13–25. 10.1007/164_2016_95 27995388

[B35] Kwon S. G. Yoon S. Y. Roh D. H. Choi S. R. Choi H. S. Moon J. Y. (2016). Peripheral neurosteroids enhance P2X receptor-induced mechanical allodynia via a sigma-1 receptor-mediated mechanism. *Brain Res. Bull.* 121 227–232. 10.1016/j.brainresbull.2016.02.012 26876754

[B36] Largent B. L. Wikstrom H. Gundlach A. L. Snyder S. H. (1987). Structural determinants of sigma receptor affinity. *Mol. Pharmacol.* 32 772–784. 2826991

[B37] Li D. Zhang S. Z. Yao Y. H. Xiang Y. Ma X. Y. Wei X. L. (2017). Sigma-1 receptor agonist increases axon outgrowth of hippocampal neurons via voltage-gated calcium ions channels. *CNS Neurosci. Ther.* 23 930–939. 10.1111/cns.12768 28990373 PMC6492695

[B38] Li H. (2017). TRP channel classification. *Adv. Exp. Med. Biol.* 976 1–8. 10.1007/978-94-024-1088-4_1 28508308

[B39] Li Z. Cui S. Zhang Z. Zhou R. Ge Y. Sokabe M. (2009). DHEA-neuroprotection and -neurotoxicity after transient cerebral ischemia in rats. *J. Cereb. Blood Flow Metab.* 29 287–296. 10.1038/jcbfm.2008.118 18854841

[B40] Li Z. Zhou R. Cui S. Xie G. Cai W. Sokabe M. (2006). Dehydroepiandrosterone sulfate prevents ischemia-induced impairment of long-term potentiation in rat hippocampal CA1 by up-regulating tyrosine phosphorylation of NMDA receptor. *Neuropharmacology* 51 958–966. 10.1016/j.neuropharm.2006.06.007 16895729

[B41] Mach R. H. Gage H. D. Buchheimer N. Huang Y. Kuhner R. Wu L. (2005). N-[18F]4’-fluorobenzylpiperidin-4yl-(2-fluorophenyl) acetamide ([18F]FBFPA): a potential fluorine-18 labeled PET radiotracer for imaging sigma-1 receptors in the CNS. *Synapse* 58 267–274. 10.1002/syn.20207 16206186

[B42] Martin W. R. Eades C. G. Thompson J. A. Huppler R. E. Gilbert P. E. (1976). The effects of morphine- and nalorphine- like drugs in the nondependent and morphine-dependent chronic spinal dog. *J. Pharmacol. Exp. Ther.* 197 517–532. 945347

[B43] Maurice T. Roman F. J. Privat A. (1996). Modulation by neurosteroids of the in vivo (+)-[3H]SKF-10,047 binding to sigma 1 receptors in the mouse forebrain. *J. Neurosci. Res.* 46 734–743. 8978508 10.1002/(SICI)1097-4547(19961215)46:6<734::AID-JNR10>3.0.CO;2-U

[B44] Maurice T. Su T. P. Parish D. W. Nabeshima T. Privat A. (1994). PRE-084, a sigma selective PCP derivative, attenuates MK-801-induced impairment of learning in mice. *Pharmacol. Biochem. Behav.* 49 859–869. 7886099 10.1016/0091-3057(94)90235-6

[B45] Maurice T. Su T. P. Privat A. (1998). Sigma1 (sigma 1) receptor agonists and neurosteroids attenuate B25-35-amyloid peptide-induced amnesia in mice through a common mechanism. *Neuroscience* 83 413–428. 9460750 10.1016/s0306-4522(97)00405-3

[B46] Mavlyutov T. A. Duellman T. Kim H. T. Epstein M. L. Leese C. Davletov B. A. (2016). Sigma-1 receptor expression in the dorsal root ganglion: Reexamination using a highly specific antibody. *Neuroscience* 331 148–157. 10.1016/j.neuroscience.2016.06.030 27339730 PMC5047027

[B47] Mavylutov T. Chen X. Guo L. Yang J. (2018). APEX2- tagging of Sigma 1-receptor indicates subcellular protein topology with cytosolic N-terminus and ER luminal C-terminus. *Protein Cell* 9 733–737. 28929457 10.1007/s13238-017-0468-5 PMC6053353

[B48] Mishra A. K. Mavlyutov T. Singh D. R. Biener G. Yang J. Oliver J. A. (2015). The sigma-1 receptors are present in monomeric and oligomeric forms in living cells in the presence and absence of ligands. *Biochem. J.* 466 263–271. 10.1042/BJ20141321 25510962 PMC4500508

[B49] Monnet F. P. Debonnel G. Junien J. L. De Montigny C. (1990). N-methyl-D-aspartate-induced neuronal activation is selectively modulated by sigma receptors. *Eur. J. Pharmacol.* 179 441–445. 2163857 10.1016/0014-2999(90)90186-a

[B50] Morales-Lazaro S. L. Llorente I. Sierra-Ramirez F. Lopez-Romero A. E. Ortiz-Renteria M. Serrano-Flores B. (2016). Inhibition of TRPV1 channels by a naturally occurring omega-9 fatty acid reduces pain and itch. *Nat. Commun.* 7:13092. 10.1038/ncomms13092 27721373 PMC5062500

[B51] Morales-Lazaro S. L. Simon S. A. Rosenbaum T. (2013). The role of endogenous molecules in modulating pain through transient receptor potential vanilloid 1 (TRPV1). *J. Physiol.* 591 3109–3121. 10.1113/jphysiol.2013.251751 23613529 PMC3717213

[B52] Mueller B. H. II Park Y. Daudt D. R. III Ma H. Y. Akopova I. Stankowska D. L. (2013). Sigma-1 receptor stimulation attenuates calcium influx through activated L-type voltage gated calcium channels in purified retinal ganglion cells. *Exp. Eye Res.* 107 21–31. 10.1016/j.exer.2012.11.002 23183135

[B53] Neill J. D. Johansson E. D. Knobil E. (1969). Patterns of circulating progesterone concentrations during the fertile menstrual cycle and the remainder of gestation in the rhesus monkey. *Endocrinology* 84 45–48. 4973116 10.1210/endo-84-1-45

[B54] Nieto-Posadas A. Picazo-Juarez G. Llorente I. Jara-Oseguera A. Morales-Lazaro S. Escalante-Alcalde D. (2011). Lysophosphatidic acid directly activates TRPV1 through a C-terminal binding site. *Nat. Chem. Biol.* 8 78–85. 10.1038/nchembio.712 22101604

[B55] Ortega-Roldan J. L. Ossa F. Schnell J. R. (2013). Characterization of the human sigma-1 receptor chaperone domain structure and binding immunoglobulin protein (BiP) interactions. *J. Biol. Chem.* 288 21448–21457. 10.1074/jbc.M113.450379 23760505 PMC3774411

[B56] Ortiz-Renteria M. Juarez-Contreras R. Gonzalez-Ramirez R. Islas L. D. Sierra-Ramirez F. Llorente I. (2018). TRPV1 channels and the progesterone receptor Sig-1R interact to regulate pain. *Proc. Natl. Acad. Sci. U.S.A.* 115 E1657–E1666. 10.1073/pnas.1715972115 29378958 PMC5816171

[B57] Pabba M. Wong A. Y. Ahlskog N. Hristova E. Biscaro D. Nassrallah W. (2014). NMDA receptors are upregulated and trafficked to the plasma membrane after sigma-1 receptor activation in the rat hippocampus. *J. Neurosci.* 34 11325–11338. 10.1523/JNEUROSCI.0458-14.2014 25143613 PMC6615506

[B58] Pal A. Hajipour A. R. Fontanilla D. Ramachandran S. Chu U. B. Mavlyutov T. (2007). Identification of regions of the sigma-1 receptor ligand binding site using a novel photoprobe. *Mol. Pharmacol.* 72 921–933. 10.1124/mol.107.038307 17622576

[B59] Palmer C. P. Mahen R. Schnell E. Djamgoz M. B. Aydar E. (2007). Sigma-1 receptors bind cholesterol and remodel lipid rafts in breast cancer cell lines. *Cancer Res.* 67 11166–11175. 18056441 10.1158/0008-5472.CAN-07-1771

[B60] Prezzavento O. Arena E. Sanchez-Fernandez C. Turnaturi R. Parenti C. Marrazzo A. (2017). (+)-and (-)-Phenazocine enantiomers: evaluation of their dual opioid agonist/sigma1 antagonist properties and antinociceptive effects. *Eur. J. Med. Chem.* 125 603–610. 10.1016/j.ejmech.2016.09.077 27721146

[B61] Ramsey I. S. Delling M. Clapham D. E. (2006). An introduction to TRP channels. *Annu. Rev. Physiol.* 68 619–647. 10.1146/annurev.physiol.68.040204.100431 16460286

[B62] Rodriguez-Munoz M. Onetti Y. Cortes-Montero E. Garzon J. Sanchez-Blazquez P. (2018). Cannabidiol enhances morphine antinociception, diminishes NMDA-mediated seizures and reduces stroke damage via the sigma 1 receptor. *Mol. Brain* 11:51. 30223868 10.1186/s13041-018-0395-2 PMC6142691

[B63] Roh D. H. Yoon S. Y. Seo H. S. Kang S. Y. Moon J. Y. Song S. (2010). Sigma-1 receptor-induced increase in murine spinal NR1 phosphorylation is mediated by the PKCalpha and epsilon, but not the PKCzeta, isoforms. *Neurosci. Lett.* 477 95–99. 10.1016/j.neulet.2010.04.041 20417251

[B64] Romero L. Merlos M. Vela J. M. (2016). Antinociception by sigma-1 receptor antagonists: central and peripheral effects. *Adv. Pharmacol.* 75 179–215. 10.1016/bs.apha.2015.11.003 26920013

[B65] Romero L. Zamanillo D. Nadal X. Sanchez-Arroyos R. Rivera-Arconada I. Dordal A. (2012). Pharmacological properties of S1RA, a new sigma-1 receptor antagonist that inhibits neuropathic pain and activity-induced spinal sensitization. *Br. J. Pharmacol.* 166 2289–2306. 10.1111/j.1476-5381.2012.01942.x 22404321 PMC3448894

[B66] Saavedra J. M. Axelrod J. (1972). Psychotomimetic N-methylated tryptamines: formation in brain in vivo and in vitro. *Science* 175 1365–1366. 5059565 10.1126/science.175.4028.1365

[B67] Sabeti J. Nelson T. E. Purdy R. H. Gruol D. L. (2007). Steroid pregnenolone sulfate enhances NMDA-receptor-independent long-term potentiation at hippocampal CA1 synapses: role for L-type calcium channels and sigma-receptors. *Hippocampus* 17 349–369. 10.1002/hipo.20273 17330865

[B68] Salazar H. Llorente I. Jara-Oseguera A. Garcia-Villegas R. Munari M. Gordon S. E. (2008). A single N-terminal cysteine in TRPV1 determines activation by pungent compounds from onion and garlic. *Nat. Neurosci.* 11 255–261. 10.1038/nn2056 18297068 PMC4370189

[B69] Schmidt H. R. Zheng S. Gurpinar E. Koehl A. Manglik A. Kruse A. C. (2016). Crystal structure of the human sigma1 receptor. *Nature* 532 527–530. 10.1038/nature17391 27042935 PMC5550834

[B70] Sha S. Qu W. J. Li L. Lu Z. H. Chen L. Yu W. F. (2013). Sigma-1 receptor knockout impairs neurogenesis in dentate gyrus of adult hippocampus via down-regulation of NMDA receptors. *CNS Neurosci. Ther.* 19 705–713. 10.1111/cns.12129 23745740 PMC6493366

[B71] Su T. P. (1982). Evidence for sigma opioid receptor: binding of [3H]SKF-10047 to etorphine-inaccessible sites in guinea-pig brain. *J. Pharmacol. Exp. Ther.* 223 284–290. 6290634

[B72] Su T. P. London E. D. Jaffe J. H. (1988). Steroid binding at sigma receptors suggests a link between endocrine, nervous, and immune systems. *Science* 240 219–221. 2832949 10.1126/science.2832949

[B73] Tam S. W. (1983). Naloxone-inaccessible sigma receptor in rat central nervous system. *Proc. Natl. Acad. Sci. U.S.A.* 80 6703–6707. 6314335 10.1073/pnas.80.21.6703 PMC391239

[B74] Tam S. W. Cook L. (1984). Sigma opiates and certain antipsychotic drugs mutually inhibit (+)-[3H] SKF 10,047 and [3H]haloperidol binding in guinea pig brain membranes. *Proc. Natl. Acad. Sci. U.S.A.* 81 5618–5621. 6147851 10.1073/pnas.81.17.5618 PMC391758

[B75] Tchedre K. T. Huang R. Q. Dibas A. Krishnamoorthy R. R. Dillon G. H. Yorio T. (2008). Sigma-1 receptor regulation of voltage-gated calcium channels involves a direct interaction. *Invest. Ophthalmol. Vis. Sci.* 49 4993–5002. 10.1167/iovs.08-1867 18641291

[B76] Tsai S. Y. Hayashi T. Mori T. Su T. P. (2009). Sigma-1 receptor chaperones and diseases. *Cent. Nerv. Syst. Agents Med. Chem.* 9 184–189. 20021352 10.2174/1871524910909030184 PMC3150837

[B77] Veerman C. C. Wilde A. A. Lodder E. M. (2015). The cardiac sodium channel gene SCN5A and its gene product NaV1.5: role in physiology and pathophysiology. *Gene* 573 177–187. 10.1016/j.gene.2015.08.062 26361848 PMC6636349

[B78] Waterhouse R. N. Chang R. C. Atuehene N. Collier T. L. (2007). In vitro and in vivo binding of neuroactive steroids to the sigma-1 receptor as measured with the positron emission tomography radioligand [18F]FPS. *Synapse* 61 540–546. 10.1002/syn.20369 17447254

[B79] Wu L. Nishiyama K. Hollyfield J. G. Wang Q. (2002). Localization of Nav1.5 sodium channel protein in the mouse brain. *Neuroreport* 13 2547–2551. 10.1097/01.wnr.0000052322.62862.a5 12499865 PMC1579862

[B80] Wu Z. Y. Yu D. J. Soong T. W. Dawe G. S. Bian J. S. (2011). Progesterone impairs human ether-a-go-go-related gene (HERG) trafficking by disruption of intracellular cholesterol homeostasis. *J. Biol. Chem.* 286 22186–22194. 10.1074/jbc.M110.198853 21525004 PMC3121363

[B81] Xu Q. Ji X. F. Chi T. Y. Liu P. Jin G. Chen L. (2017). Sigma-1 receptor in brain ischemia/reperfusion: possible role in the NR2A-induced pathway to regulate brain-derived neurotrophic factor. *J. Neurol. Sci.* 376 166–175. 10.1016/j.jns.2017.03.027 28431607

[B82] Yabuki Y. Shinoda Y. Izumi H. Ikuno T. Shioda N. Fukunaga K. (2015). Dehydroepiandrosterone administration improves memory deficits following transient brain ischemia through sigma-1 receptor stimulation. *Brain Res.* 1622 102–113. 10.1016/j.brainres.2015.05.006 26119915

[B83] Zhang H. Cuevas J. (2002). Sigma receptors inhibit high-voltage-activated calcium channels in rat sympathetic and parasympathetic neurons. *J. Neurophysiol.* 87 2867–2879. 10.1152/jn.2002.87.6.2867 12037190

[B84] Zhang K. Zhao Z. Lan L. Wei X. Wang L. Liu X. (2017). Sigma-1 receptor plays a negative modulation on N-type calcium channel. *Front. Pharmacol.* 8:302. 10.3389/fphar.2017.00302 28603497 PMC5445107

[B85] Zhu S. Wang C. Han Y. Song C. Hu X. Liu Y. (2015). Sigma-1 receptor antagonist BD1047 reduces mechanical allodynia in a rat model of bone cancer pain through the inhibition of Spinal NR1 phosphorylation and microglia activation. *Mediators Inflamm.* 2015:265056. 10.1155/2015/265056 26696751 PMC4677253

[B86] Zou L. B. Yamada K. Sasa M. Nakata Y. Nabeshima T. (2000). Effects of sigma(1) receptor agonist SA4503 and neuroactive steroids on performance in a radial arm maze task in rats. *Neuropharmacology* 39 1617–1627. 10854906 10.1016/s0028-3908(99)00228-2

